# The Effect of Polyethylene Fiber and Flowable Resin Composite on Fracture Resistance in the Restoration of Large MOD Cavities

**DOI:** 10.1111/jerd.13413

**Published:** 2025-01-18

**Authors:** Gökhan Karadağ, Betül Erdal

**Affiliations:** ^1^ Department of Restorative Dentistry, Faculty of Dentistry Kırıkkale University Kırıkkale Türkiye

**Keywords:** fiber positions, fracture resistance, polyethylene fibers, resin composites, short fibers

## Abstract

**Objective:**

This in vitro study aims to evaluate the effect of placing polyethylene fibers used in large Class II MOD (mesio‐occlusion‐distal) cavities into different flowable resin composites and in different positions on the fracture resistance of the restoration.

**Materials and Methods:**

Ninety healthy human molars were used in the study. No treatment was performed on 10 of these teeth and they were used as the control group. The other 80 teeth were divided into 8 groups by opening large MOD cavities (*n* = 10). The groups were divided into 4 main groups according to the use of fiber strips; no fiber, placed in the buccolingual direction, placed in the mesiodistal direction, and placed in an “O” shape on all walls. Each main group was divided into two subgroups according to the use of the nanofill flowable composite or flowable short fiber resin composite (SFRC) as the base material. All teeth were aged with a thermal cycler and then fractured with a universal testing machine. Fracture types were recorded in terms of fracture strength and repairability. Kruskal–Wallis, one‐way ANOVA, and Tukey post hoc tests were used to compare fracture strength values (*p* < 0.05).

**Results:**

The highest fracture resistance was observed in the control group (2888.67 ± 395.43 N). The control group was significantly higher than all groups except the 7th and 8th groups (*p* < 0.05). Among the restored groups, the highest mean fracture resistance values were observed in the 8th group (2463.92 ± 332.37 N). The 8th group was statistically significantly higher than the 1st and 2nd groups (*p* < 0.05). There was no statistically significant difference between any of the other restored groups (*p* > 0.05).

**Conclusions:**

The application of polyethylene fiber in the treatment of teeth with large MOD cavities in different positions did not result in a significant difference in fracture resistance.

## 1 Introduction

Although resin composites were previously preferred mostly in anterior teeth, today they are also frequently preferred in posterior teeth with the increase in aesthetic expectations and developments in resin composites [1, 2]. However, it has been shown that its use does not provide the desired success rate, especially in multisurface large complex posterior cavities [3, 4]. Continuous studies are carried out to improve the physical and mechanical properties of resin composites and to prevent failures in restorations, and different materials are introduced to the market [5, 6]. One of the preferred methods for this purpose is the use of fiber‐reinforced composites in dentistry [7, 8]. Fiber‐reinforced composite restorations are resin‐based restorations that incorporate fibers for the purpose of enhancing the physical properties of the resin [9].

Four main types of fiber are used in dentistry: carbon, aramid, polyethylene, and glass fiber [10, 11]. Polyethylene fiber, developed by Cappacio and Ward in 1973, is a natural crystalline polymer [12]. Polyethylene fibers enhance the impact strength, elastic modulus, and flexural strength of composite materials. However, they are virtually invisible in a resin matrix, making them an optimal aesthetic reinforcer of composite materials [13].

In 1992, Ribbond (Ribbond Inc., Seattle, WA) was introduced to the market as a bondable reinforced fiber comprising ultrahigh‐strength polyethylene fibers. The Ribbond's patented leno weave feature ensures that the forces are transferred effectively throughout the weaving process, obviating the need to transfer the stress back to the resin [14]. It has been reported that the use of the ribbond in large MOD cavities has been shown to increase coronal resistance [15]. Different fiber‐reinforced composite restoration techniques are introduced, such as placing the ribbond under the composite restoration or placing a cavity over the finished composite restoration [15, 16]. Also, it is reported that the placement of the fibers to be used to increase the resistance of the coronal restoration in the cavity may affect the performance of the applied procedure [17].

In 2013, short fiber reinforced resin composite (SFRC) (everX Posterior; GC, Tokyo, Japan) was introduced to the market to replace the lost dentin with a material with similar mechanical properties, especially in posterior teeth in areas exposed to high chewing force [18]. Due to the high viscosity of everX Posterior, the manufacturer launched a fluid form with lower viscosity in 2019 (everX Flow; GC, Tokyo, Japan) [19].

This in vitro study aims to evaluate the effect of placing polyethylene fibers used in large Class II mesial‐occlusion‐distal (MOD) cavities in different positions within the SFRC on the fracture resistance of the restoration.

The null hypotheses are as follows:

1. Placing polyethylene fiber in different positions in the cavity has no effect on fracture strength.
2. Placing polyethylene fiber in different resin composites has no effect on fracture strength.

## 2 Material Methods

### 2.1 Selection and Preparation of Samples

Approval for this study was obtained from the Non‐Interventional Research Ethics Committee of Kırıkkale University (Decision number: 2023.03.23). In the study, 90 sound human molar teeth extracted for periodontal or orthodontic reasons with similar sizes and shapes were used. After extraction, the teeth were cleaned of debris and disinfected with 0.5% chloramine‐T solution for 7 days. They were then stored in +4°C distilled water until use. The teeth were kept in distilled water for a maximum of 3 months. The roots were embedded vertically in self‐cure acrylic resin (Imicryl SC; Imicryl Dental Materials Inc., Konya, Turkey) using standard molds, 2 mm below the cementoenamel (CEJ) junction.

**FIGURE 1:**
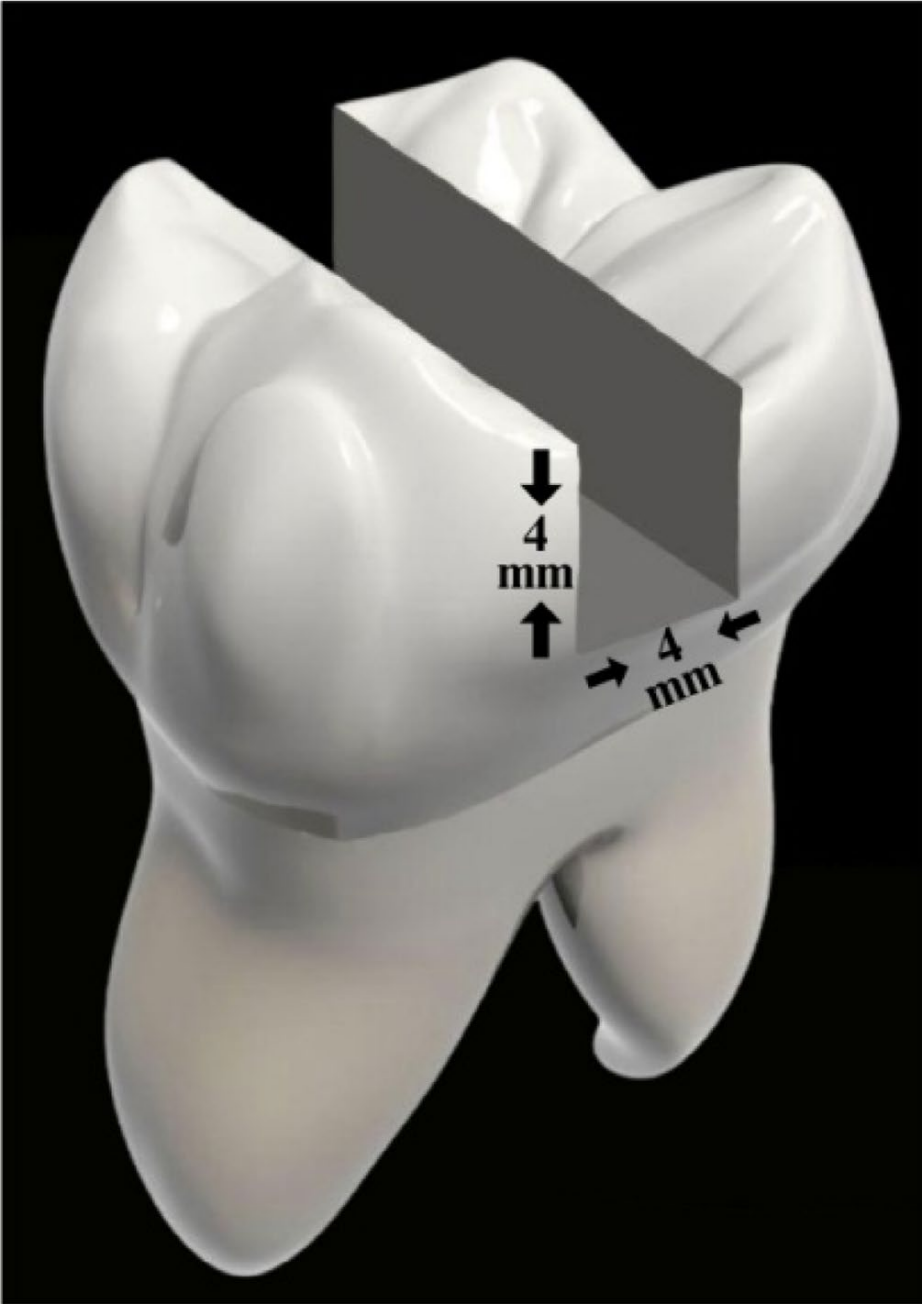
Cavity design.

Standard MOD cavities with occlusogingival and buccolingual widths of 4 mm were drilled in 80 of the prepared specimens (Figure 1). The cavities were prepared with an aerator under water cooling using diamond fissure burs. A periodontal probe was used to measure the cavity dimensions.

### 2.2 Restorative Procedures

No procedure was performed on 10 unprepared intact teeth and these were considered as the control group. The 80 prepared teeth were randomly divided into eight groups with 10 teeth in each group. Matrix bands (SuperMat, Kerr, Orange, CA) were applied to the specimens. The enamel surfaces were selectively etched for 30 s, rinsed, and then the universal adhesive system (G Premio Bond, GC, Tokyo, Japan) was used as an adhesive according to the manufacturer's instructions. The adhesive was polymerized with an LED device (Ellipar, 3M/ESPE, St. Paul, MN) in standard mode with 1200 mW/cm^2^ for 20 s. First, the mesial and distal proximal surfaces of the cavities were formed with a microhybrid resin composite (Genial Posterior, GC, Tokyo, Japan) and polymerized for 20 s each. Thus, standard class 1 cavities were obtained. These procedures were performed in the same way for all groups. Afterwards, different restorative procedures were performed for each group.

**FIGURE 2:**
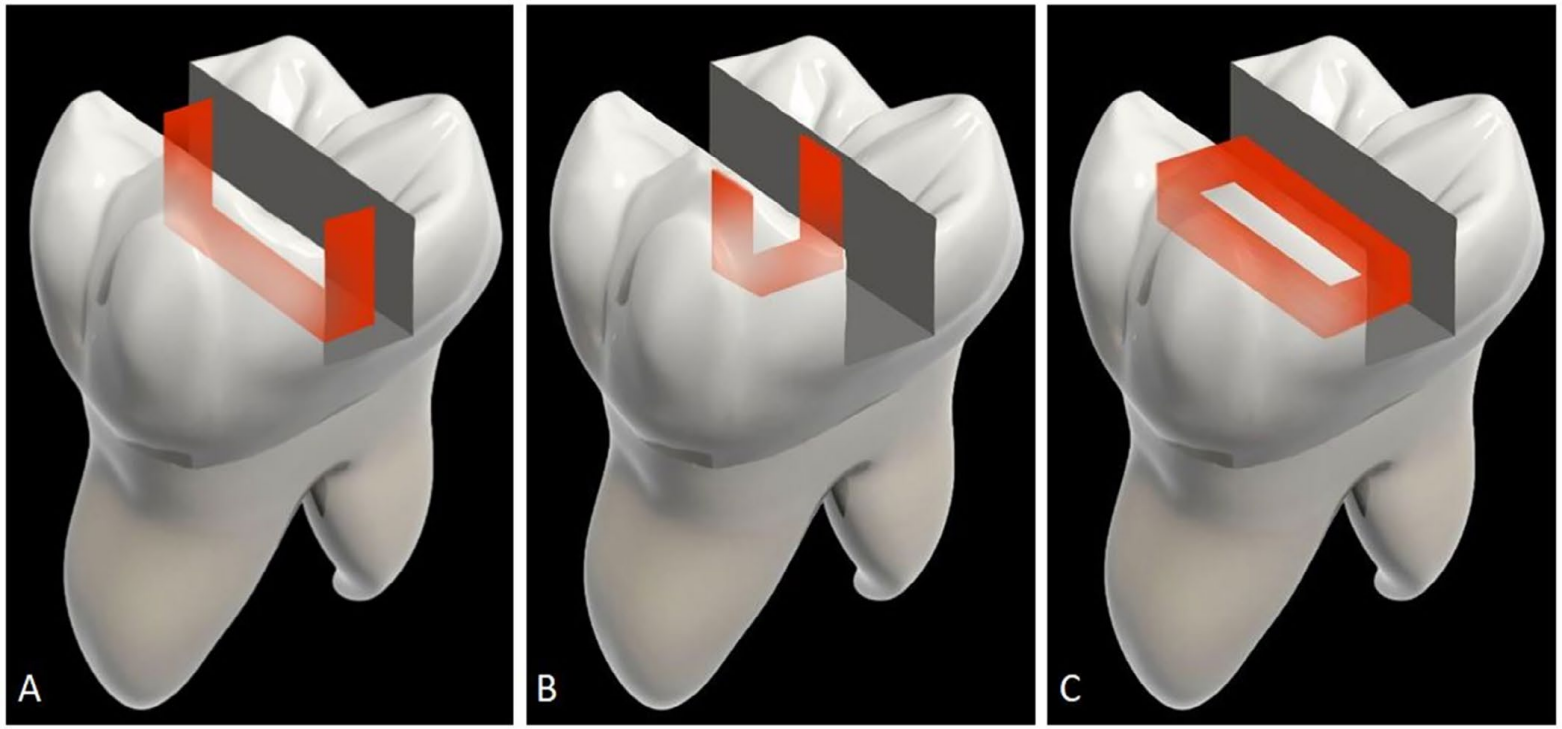
Ribbond configurations. (A) Mesial‐occlusal‐distal. (B) Buccal‐occlusal‐lingual. (C) Buccal‐mesial‐lingual‐distal.

Different restorative procedures were applied to different groups in the first 2 mm of Class 1 cavities, as indicated below (Figure 2). All restorative materials used in the study are listed in Table 1.

- Group 1 (NFR): Only nanofilled flowable resin composite was placed.
- Group 2 (SFRC): Only flowable short fiber resin composite was placed.
- Group 3 (NFR+MOD): The fiber strip was integrated into the nanofilled flowable resin composite in a “U” configuration, in direct contact with the mesial‐occlusal‐distal walls.
- Group 4 (SFRC+MOD): The fiber strip was integrated into the flowable short fiber resin composite in a “U” configuration, in direct contact with the mesial‐occlusal‐distal walls.
- Group 5 (NFR+BOL): The fiber strip was integrated into the nanofilled flowable resin composite in a “U” configuration, in direct contact with the buccal‐occlusal‐lingual walls.
- Group 6 (SFRC+BOL): The fiber strip was integrated into the flowable short fiber resin composite in a “U” configuration, in direct contact with the buccal‐occlusal‐lingual walls.
- Group 7 (NFR+BMLD): The fiber strip was integrated into the nanofilled flowable resin composite in an “O” configuration, in direct contact with the buccal‐mesial‐lingual‐distal walls.
- Group 8 (SFRC+BMLD): The fiber strip was integrated into the flowable short fiber resin composite in an “O” configuration, in direct contact with the buccal‐mesial‐lingual‐distal walls.

**TABLE 1:** The composition of materials used in the study.

Material	Manufacturer	Composition	Lot number
G‐Aenial posterior Microhybrid resin composite	GC Tokyo, Japan	UDMA, dimethacrylate co‐monomers, prepolymerized silica, and strontium fluoride containing fillers 80 wt%	230323A
Clearfil majesty flow Nanofilled flowable resin composite	Kuraray Tokyo, Japan	TEGDMA, hydrophobic aromatic dimethacrylate, silanated barium glass filler, silanated silica filler, dl‐camphorquinone, accelerators, initiators, and pigments. Filler size: 0.18–3.5 mm	3E0032
everX flow Flowable short fiber resin composite	GC Tokyo, Japan	Bis‐EMA, TEGDMA, UDMA, micrometer scale glass fiber filler, Barium glass 70 wt%, 46 vol%	2311141
Ribbond Polyethylene fiber	Ribbond Seattle, WA, USA	Preimpregnated, silanized, plasma‐treated, leno‐woven, ultrahigh‐modulus polyethylene fibers	+D758T0/$$7350/16D20221212/4
G‐Premio bond Self‐etch adhesive system	GC Tokyo, Japan	10‐MDP, 4‐META, 10‐methacryoyloxydecyl dihydrogen thiophosphate, methacrylate acid ester, distilled water, acetone, photo‐initiators, silica fine powder	2305091
Ribbond wetting resin	Ribbond Seattle, WA, USA	Methacrylate monomers, photoinitiators, stabilizers	676411

Abbreviations: 10‐MDP, 10‐metakriloloksidesil dihidrojenfosfat; 4‐META, 4‐metakriloloksietil trimellitat anhidrat; Bis‐EMA, ethoxylated bisphenol A‐dimethacrylate; TEGDMA, triethylene glycol dimethacrylate; UDMA, urethane dimethacrylate.

In the groups where Ribbond was used (Group 3–8), fiber strips were cut to the desired lengths with specific scissors of the Ribbond kit. It was soaked in a bonding agent adhesive resin (Ribbond Wetting Resin, Ribbond, Seattle, WA), and excess material was dried with gauze. The strip was then embedded in the composite resin at the positions indicated in the groups.

For all groups, the first layers were polymerized for 20 s. Then the final layer of 2 mm was restored with G‐Aenial Posterior in all groups and polymerized for 20 s. The restorations were finished and polished using 3M Sof‐Lex disks (Dental products/3 M, St. Paul, MN). The resulting specimens were soaked in distilled water at 37°C for 7 days before aging by thermal cycling.

### 2.3 Aging and Fracture Resistance Test

A thermocycling machine (Esetron, Mod Dental, Ankara, Turkey) was used to mimic oral conditions. All samples were subjected to 10,000 thermal cycles between 5°C and 55°C, with a dwell time of 30 s and a transfer time of 10 s.

**FIGURE 3:**
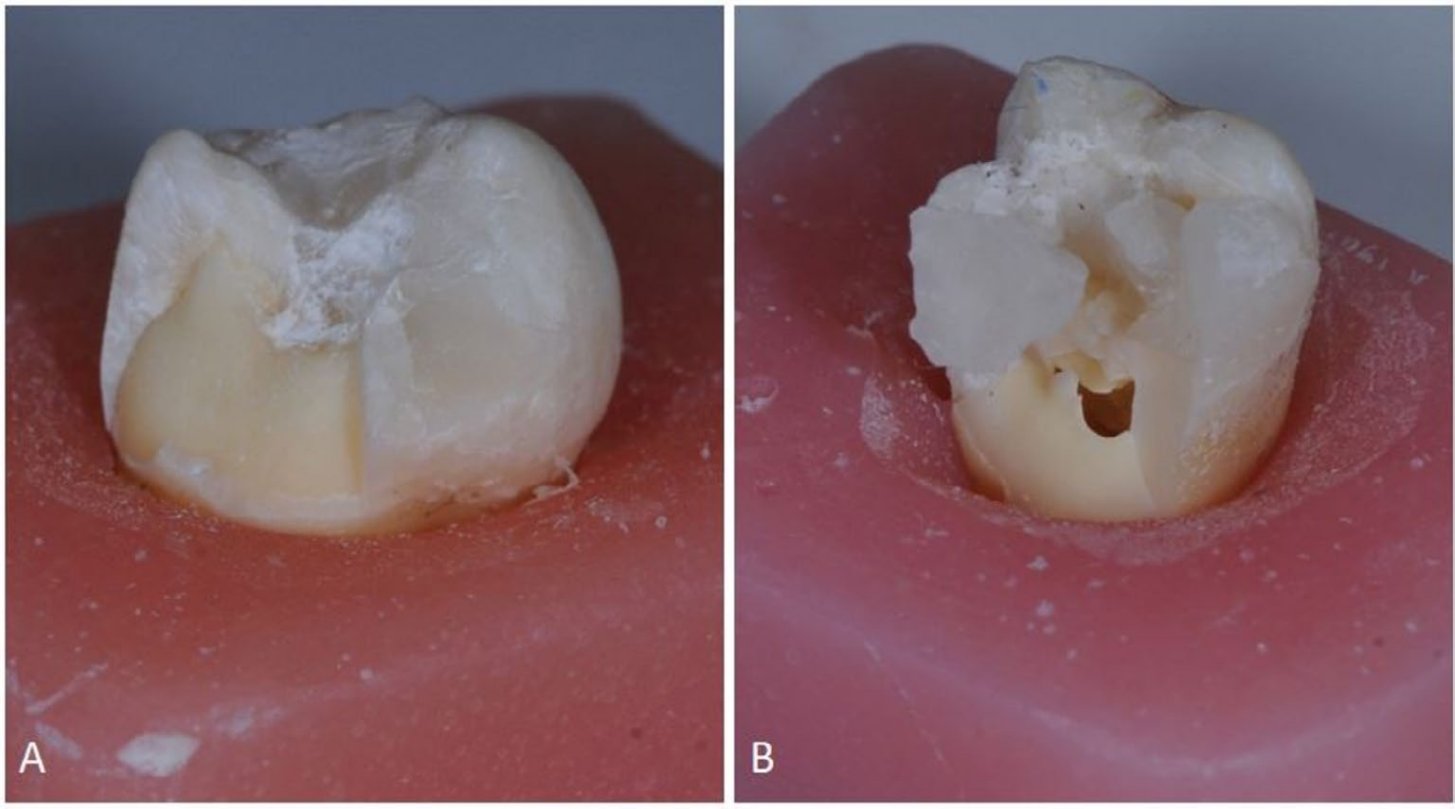
Fracture types. (A) Repairable. (B) Unrepairable.

All specimens were placed in an Instron universal testing machine with the test tip perpendicular to the occlusal fossa. The force was applied at a crosshead speed of 0.5 mm/min until fracture occurred. The force at which the fracture occurred was recorded in newtons (N). A visual distinction was made between the two fracture modes, with consideration given to the repairability of the tooth. Fractures up to 1 mm below the DEJ were classified as “repairable,” while fractures more than 1 mm below the DEJ were classified as “unrepairable” (Figure 3).

### 2.4 Statistical Analysis

SPSS (version 27.0; IBM, Armonk, NY) was used for statistical analysis. The data were examined for normal distribution using the Shapiro–Wilk test. All parameters showed deviation from normal distribution, so nonparametric tests were conducted. Kruskal–Wallis, one‐way ANOVA and Tukey post hoc test were used (*p* < 0.05).

## 3 Results

Table 2 shows the mean load at fracture values, standard deviations, and statistical comparisons for each group. The highest fracture resistance was observed in the control group (2888.67 ± 395.43 N). The control group was significantly higher than all groups except the 7th and 8th groups (*p* < 0.05). Among the restored groups, the highest mean fracture resistance values were observed in the 8th group (2463.92 ± 332.37 N). The 8th group was statistically significantly higher than the 1st and 2nd groups (*p* < 0.05). There was no statistically significant difference between any other restored groups (*p* > 0.05).

**TABLE 2:** Mean fracture resistance for all groups.

Groups	*n*	Mean ± SD (*N*)	Std. error	Minimum	Maximum	*p*
Control	10	2888,67 ± 395,43^a^	125,04	2025,27	3458,83	—
Group 1	10	1933,67 ± 202,63^b^	64,07	1620,12	2287,44	0,000
Group 2	10	1964,84 ± 207,58^b^	65,64	1639,38	2292,36	0,001
Group 3	10	2027,35 ± 219,41^bc^	69,38	1760,20	2496,32	0,001
Group 4	10	2216,16 ± 292,92^bc^	92,63	1799,61	2578,46	0,017
Group 5	10	1900,43 ± 349,67^bc^	110,57	1552,27	2511,33	0,001
Group 6	10	1942,16 ± 325,15^bc^	102,82	1426,39	2527,32	0,001
Group 7	10	2379,02 ± 348,85^abc^	110,31	1896,74	2936,90	0,220
Group 8	10	2463,92 ± 332,37^ac^	105,10	1948,97	3008,31	0,487
Total	90	2190,69 ± 429,12	45,23	1426,39	3458,83	

*Note*: Different superscript letter indicates statistically significant difference in the rows.

Most unrepairable fractures were observed in the control group (10%), while most repairable fractures were observed in the second group (70%) (Table 3). Examples of repairable and unrepairable fracture types are shown in Figure 3.

**TABLE 3:** Number and percentage of fracture modes of groups.

Groups	Repairable	Unrepairable
Control	1 (10%)	9 (90%)
Group 1	3 (30%)	7 (30%)
Group 2	7 (70%)	3 (30%)
Group 3	1 (10%)	9 (90%)
Group 4	5 (50%)	5 (50%)
Group 5	3 (30%)	7 (70%)
Group 6	4 (40%)	6 (60%)
Group 7	3 (30%)	7 (70%)
Group 8	2 (20%)	8 (80%)
Total	29 (32%)	61 (68%)

## 4 Discussion

The use of fibers in the treatment of teeth with large defects is often preferred today. In this in vitro study, we aimed to investigate the effects of the application of polyethylene fibers in molar teeth with large MOD cavities, the fiber position, and the placement of the fiber in different resin composites on fracture resistance. The placement of polyethylene fibers in different positions did not produce a statistically significant difference in fracture resistance (*p* > 0.05). Placement of polyethylene fibers in a flowable short fiber resin composite instead of a nanofilled flowable composite showed a positive effect on fracture resistance, but this effect did not create a significant difference (*p* > 0.05). Therefore, both null hypotheses were accepted in our study.

Many studies have shown that the use of polyethylene fiber in teeth with large cavities has a positive effect on fracture resistance [15, 20, 21]. In our study, the 8th group, in which the polyethylene fiber was placed in the “O” configuration in the flowable short fiber resin composite, showed higher fracture resistance than the 1st and 2nd groups, in which no polyethylene fiber was used. There was no statistically significant difference among the other experimental groups. These results are partially consistent with other studies. We believe that the reason why the use of polyethylene fiber has a limited effect on fracture resistance in our study may be the cavity design and dimensions we used in the study. The effect of the polyethylene fiber may be more significant in larger cavities with less remaining tooth tissue.

One of the curious issues about fibers is the effect of different shapes and positions on fracture strength. Nevertheless, the number of studies on this subject is limited, and the preferred fiber positions in these studies are not standard. Therefore, it is very difficult to reach a clear idea. Some studies show that the application of fibers in different positions has an effect on fracture resistance [22, 23, 24]. However, Ramírez‐Gómez et al. [25] used polyethylene fibers in different positions in the restoration of standard MOD and occluso‐buccal cavities prepared in extracted premolars. In this study, the application of the fiber to the cavity was conducted in four different positions: horizontal, vertical, bidirectional, and circular. The results indicated that the fracture strength was not statistically significantly different between the four positions. Similarly, our study found no statistically significant difference in fracture strength between the different fiber positions. These findings are consistent with those of Ramírez‐Gómez et al., who employed a similar methodology, including the same cavity design, fiber material, and positions.

Our study also investigated the effect of SFRCs on fracture resistance compared with nanofill composites. The beneficial effect of the use of SFRCs on fracture strength in large cavity restorations is well known [26]. It has also been reported that this material, in addition to its effect on fracture resistance, influences the fracture type and contributes to a more repairable fracture type [27]. However, it has been reported in the literature that in some studies SFRCs may only minimally increase the fracture resistance of restorations and that this increase may not be statistically significant. This is related to the effect of the area where the fibers are placed on the stress distribution of the restoration. It has been reported that fibers increase fracture resistance, especially in large and deep cavity restorations, but this effect is less pronounced in small or shallow cavities [28]. According to the results of our study, Group 2 with SFRC showed a slightly higher mean fracture resistance than Group 1 with nanofilled flowable resin composite, but this difference was not statistically significant. However, in terms of repairable fracture type, Group 2 (70%) showed a significantly higher rate than Group 1 (30%). We can say that these results are in line with the literature.

Although there are many studies investigating the use of SFRC and polyethylene fiber in the restoration of large cavities, there are very few studies in the literature investigating the combined use of SFRC and polyethylene fiber. Soto‐Cadena et al. [29] used polyethylene fiber, SFRC and SFRC in combination for the restoration of standard MOD cavities in extracted premolars. According to their results, they reported the highest fracture resistance in the group in which SFRC and polyethylene fiber were used in combination. In our study, although the groups in which we used SFRC and polyethylene fiber in combination showed higher mean fracture resistance than the groups restored with polyethylene fiber alone, this difference was not statistically significant (*p* > 0.05). Therefore, the results of our study are partially compatible with the study by Soto‐Cadena et al. The reason for the limited effect of combined use in our study may be the MOD cavity dimensions and the fact that we preferred molars instead of premolars. Because it is known that premolars are more prone to cuspal fractures against destructive forces than molars [30]. Another difference between these two studies is that in our study, we achieved combined use by placing the polyethylene fiber in the SFRC, whereas Soto‐Cadena et al. obtained combined use by placing the polyethylene fiber in a conventional flowable composite and applying SFRC on it. This may have caused the difference between the groups to become apparent.

The specimens we prepared from extracted teeth in the current study do not mimic oral tissues in the same way. However, it is possible that the force applied perpendicular to the specimens to measure fracture resistance did not simulate the dynamic loading conditions created by biting and chewing forces. These can be considered the major limitations of the current study. Therefore, it is recommended that the data obtained from the study be supported by long‐term clinical trials.

## 5 Conclusions

Within the limitations of the study, the following results were obtained in this in vitro study:

1. The application of polyethylene fiber in the treatment of teeth with large MOD cavities in different positions did not result in a significant difference in fracture resistance.
2. The placement of polyethylene fiber in flowable short fiber resin composite, as opposed to nanofilled flowable resin composite, had a positive effect on fracture resistance; however, it did not make a significant difference.

## Conflicts of Interest

The authors declare no conflicts of interest.

## Data Availability

The data that support the findings of this study are available from the corresponding author upon reasonable request.
